# Mifepristone Treatment in Pregnant Murine Model Induced Mammary Gland Dysplasia and Postpartum Hypogalactia

**DOI:** 10.3389/fcell.2020.00102

**Published:** 2020-02-21

**Authors:** Hongmei Zhu, Xuchen Jia, Mingli Ren, Liguo Yang, Jianguo Chen, Li Han, Yi Ding, Mingxing Ding

**Affiliations:** College of Veterinary Medicine, Huazhong Agricultural University, Wuhan, China

**Keywords:** mammary gland dysplasia, postpartum hypogalactia, mifepristone, milk yields, hormones, murine model

## Abstract

Mammary gland dysplasia and postpartum hypogalactia often occur in humans and in the livestock breeding industry. However, their underlying mechanisms are not clear yet. Mifepristone, which has a high affinity for progesterone (P_4_) and glucocorticoid receptors, was exploited here to induce the disorders of mammary gland development and lactation. Four strategies were devised for treating pregnant mice with mifepristone. In the first strategy, mice were administered 1.20 mg mifepristone/kg body weight (BW) on pregnancy day 4 (Pd4). In the second strategy, mifepristone was administered to mice twice, with 1.20 mg/kg BW on Pd4 and 0.40 mg/kg BW on Pd8. In the third strategy, mice were treated with a single dose of 0.40 mg mifepristone/kg BW on Pd8. In the fourth strategy, mice were administered 0.40 mg mifepristone/kg BW on Pd8 and 0.20 mg mifepristone/kg BW on Pd12. The results suggested that mifepristone administration at the dose of 1.20 mg/kg BW on Pd4 caused significant reduction in milk production on lactation day 1 (Ld1), Ld2, and Ld3, as assessed using a weigh-suckle-weigh assay. Mammary β-casein expression, milk yields, litter growth rates, gland structure, and serum concentrations of 17-β estrogen (E_2_), P_4_, prolactin (PRL), growth hormone (GH), corticosterone (CORT) and oxytocin (OT) as well as the receptors of these hormones were determined during pregnancy or lactation after performing the first (Pd4) strategy. The results demonstrated that mifepristone administration during early pregnancy decreased β-casein expression, milk yields and litter growth rates, induced fewer alveoli, enlarged alveolar lumina, and altered the levels of E_2_, P_4_, PRL, GH, CORT, and OT as well as the mRNA expression of these hormonal receptors during pregnancy or early lactation. The present study on pregnant mice treated with mifepristone offers an innovative murine model to study the mechanism underlying mammary gland dysplasia and postpartum hypogalactia.

## Introduction

Breast milk is the first and the ideal nutritional option for infants (Czosnykowska-Lukacka et al., 2018). The insufficient production of colostrum during the first 3 days of lactation after parturition can seriously affect the nutrition and the growth of infants. In pigs, the syndrome accompanied with a primary clinical sign of the sow’s inability to produce sufficient milk to meet the nutritional requirements of piglets was named as postpartum dysgalactia syndrome (PDS) or postpartum hypogalactia syndrome (PHS). These syndromes in sows are observed almost within the first 3 days postpartum and can cause decreased growth performance, depression, or diarrhea in piglets (Marchant et al., 2000; Bilkei, 2005; Klopfenstein et al., 2006; Preissler et al., 2012). In cows and goats, these phenomenon are called early hypogalactia, which could induce the malnutrition or even death of their neonates. Nowadays, more attention is being paid to postpartum hypogalactia, due to its harm to the neonates and the breeding industry. Some studies have explored the mechanisms of postpartum hypogalactia. Milk insufficiency in mothers of hospitalized infants is often blamed on stress, which interferes with the release of oxytocin (OT) and milk ejection reflex (Nissen et al., 1998). In pig herds, factors that influence the occurrence of hypogalactia or agalactia include infectious diseases (e.g., mastitis or metritis) (Backstrom et al., 1984; Kemper and Gerjets, 2009), stress (e.g., abrupt transfer of dams) (Bäckström et al., 1984), poor management (e.g., parturition induction, feed allowance or nutritional imbalance) (Goransson, 1989; Foisnet et al., 2011), or endocrine disorders (Foisnet et al., 2010). However, all the factors above are likely to disturb the entire endocrine system, which may adversely affect the mammary gland development and lactation.

Mice are a good model for studying mammary gland development and lactation. Several hormones play a key role in regulating mammary gland development and lactation in mice (Neville et al., 2002; Arendt and Kuperwasser, 2015). The reproductive and metabolic hormones, such as estrogen, progesterone (P_4_), placental lactogen, prolactin (PRL), OT, growth hormone (GH), glucocorticoids, thyroid hormone and insulin, well coordinate and dominate mammary gland development including ductal branching, alveolar bud development and secretion during pregnancy or lactation (Neville et al., 2002; Macias and Hinck, 2012; McNally and Stein, 2017). Estrogen and GH primarily regulate ductal morphogenesis by binding to their receptors during puberty and pregnancy in mice (Kleinberg et al., 2000; McBryan and Howlin, 2017). A balance between P_4_ and PRL controls alveolar morphogenesis during pregnancy in mice (Neville et al., 2002; Leehy et al., 2018), while PRL, GH, glucocorticoids, thyroid hormone, and insulin govern milk secretion in mice or rats (Vonderhaar and Ziska, 1989; Flint and Gardner, 1994; Menzies et al., 2010). Nevertheless, any imbalance in the levels of these hormones may be closely related to problems in mammary gland growth or milk secretion in domestic animals (Barnes et al., 1985). For instance, a previous study has reported greater plasma concentrations of P_4_ and less of PRL in sows that produced a low yield of colostrum compared to those produced a high yield of colostrum before parturition (Foisnet et al., 2010). A lower PRL/P_4_ ratio 24 h pre-partum, characterized by a higher P_4_ concentration and a lower PRL concentration peripartum, led to the decreased colostrum yield in primiparous sows, and vice versa (Loisel et al., 2015). Higher concentrations of serum cortisol were reported in sows with mastitis metritis agalactia (MMA) syndrome up to day 10 postpartum than that in healthy sows (Bilkei, 2005).

Mifepristone (RU486) is a synthetic steroid with a strong affinity to P_4_ receptor (PR) and glucocorticoid receptor (GR) and, to a lesser extent, to the androgen receptor (AR) (Philibert et al., 1985). However, mifepristone interacts with PR and GR in a different manner than P_4_, occupying the receptor without stimulating P_4_-related gene transcription (Spitz and Bardin, 1993). It has been reported that short-term administration of mifepristone decreased luteinizing hormone (LH) in the follicular and luteal phases of the cycle, whereas long-term administration of mifepristone (i.e., 3 months) increased the secretion of LH in human (Shoupe et al., 1987; Garzo et al., 1988; Permezel et al., 1989; Kettel et al., 1991). Researchers found that mifepristone stimulated the secretion of PGF_2α_ and reduced its metabolism both *in vitro* and *in vivo* (Smith and Kelly, 1987; Norman et al., 1991). As PGF_2α_ could induce functional regression and cell apoptosis of the corpus luteum in rodents (Wang et al., 2003), and could reduce the number of primary (growing) follicles of pregnant mice both *in vivo* and *in vitro* (Peluso et al., 1980), this may directly decrease serum levels of P_4_ and E_2_ after short-term mifepristone administration. Since being discovered in 1980, mifepristone has generated immense interest in clinical application and in research. Until now, the clinical use of mifepristone was mainly confined to termination of early pregnancy (Couzinet et al., 1986; Birgerson and Odlind, 1987; Spitz et al., 1998; Sonalkar et al., 2019). Lately, mifepristone is being extensively tested for induction of labor (Jindal et al., 2019), treatment of endometriosis or uterine leiomyomata (Kettel et al., 1996; Eisinger et al., 2003), ovarian or prostate cancer (Goyeneche et al., 2007; Ligr et al., 2012), Cushing’s syndrome and major psychotic depression (Blasey et al., 2011). Toxicology studies on animal exposure up to 6 months suggested that mifepristone had no mutagenic potential and no toxic effect up to 1000 mg/kg dose in acute administration in several species (Baulieu, 2013). In sub-chronic toxicity studies in rodents and monkeys, daily doses of mifepristone up to 200 or 125 mg/kg BW displayed no toxicity but antihormonal effects. For example, the anti-progesterone effects were frequent estrus, decrease in uterine weight, inadequate mammary development, suppression of menstruation, and less serum progesterone in monkeys; anti-glucocorticoid effects included increased kidney and adrenal weights in rats and monkeys as well as higher serum concentrations of adrenocorticotropic hormone (ACTH) and cortisol in monkeys; while, anti-androgenic effects included a lower weight of prostates and seminal vesicles weights in male rats. However, the surviving fetuses of rats and mice showed no anomaly when mifepristone was administered at a sub-abortive dosage (Sitruk-Ware and Spitz, 2003; Baulieu, 2013).

It is difficult to retrospectively investigate the changes in hormone profiles during gestation or lactation, and to investigate their impact on mammary gland development and lactation in animals. Therefore, to clarify the mechanisms underlying mammary gland dysplasia and postpartum hypogalactia, it is imperative to establish an animal model, in which the endocrine system is disrupted during pregnancy and early lactation. Several murine hormone receptor knock-out models (e.g., receptors of 17β-estrodiol (E_2_), P_4_, and glucocorticoids) were used for studying the functions of hormones and their receptors. However, these gene knock-out animals exhibited significant defects in multiple tissues (e.g., ovary, uterus, mammary gland, or lung), or had extremely low survivals rates (Cole et al., 1995; Lydon et al., 1995; Bocchinfuso and Korach, 1997). Since mifepristone treatment during early pregnancy decreased the levels of 17 β-estradiol (E_2_) and P_4_ in mice and humans (Chen et al., 2015; Baev et al., 2017), it was exploited in the present study to disturb the hormone balance, and to establish a murine model for mammary gland dysplasia and postpartum hypogalactia. The milk yields, pup growth, mammary histology, serum hormones, and hormone receptors were assessed to evaluate the model and to explain the mechanisms of milk insufficiency after mifepristone treatment. This murine model would be valuable in investigating how alterations in several hormones lead to hypogalactia during early lactation in livestock.

## Materials and Methods

### Animals

Specific-pathogen-free (SPF) grade Kunming female virgin mice aged approximately 7 weeks (Laboratory Animal Quality Certificate Number: 42000600031575) were provided by Hubei Provincial Center for Laboratory Animal Research, Wuhan, Hubei Province, China. These mice were housed in a temperature-controlled (22 ± 2°C) room with a relative humidity of 50–70%. They received food pellets and water *ad libitum*. One week was allowed for their adaption to the surrounding environment. Female mice (31 ± 1 g) were mated with males, and the mating was confirmed by the presence of a vaginal plug. The day when the vaginal plugs were observed, was counted as day 1 of pregnancy (Pd1), and the day following the parturition, was considered as day 1 of lactation (Ld1). Females were caged individually during the perinatal period. All experimental procedures were approved by Hubei Provincial Center for Laboratory Animal Research, and were performed strictly in accordance with the guidelines of Institutional Animal Care and Use Committee of Huazhong Agricultural University.

### Experimental Groups and Treatments

Mifepristone (≥95% purity) was purchased from Beijing Solarbio Science & Technology Co., Ltd. (Beijing, China), and was dissolved in 1, 3-propanediol to prepare a stock solution of 0.01 mg/mL. A previous study in mice reported that the ducts from the nipples and buds were beginning to appear along the smaller ducts on Pd2 or Pd3; on Pd6, alveoli formation was taking place along all the ducts; from Pd7 to Pd12, the growth and budding of mammary gland proceed steadily, and the alveoli developed thickly along ducts until Pd12 (Cole, 1933). Researchers verified that administration of mifepristone at doses of 1.25–2.50 mg/kg BW on Pd4 had little effect on the litter size in mice (Huang et al., 2005; Lv et al., 2012; Chen et al., 2015), whereas when mifepristone was administrated at doses of 0.30–2.00 mg/kg and 0.401–2.50 mg/kg, respectively, on Pd8.5 and on Pd14–19, it induced more than 60% of abortions (Szekeres-Bartho et al., 1997; Lv et al., 2012). Based on the time points of mammary gland development, and the dose range of mifepristone that induce abortion in the studies described above, we administrated mifepristone on Pd4 (0.80, 1.20, 1.60, 2.00, and 2.40 mg/kg BW), Pd8 (0.20, 0.40, 0.60, 0.80, and 1.00 mg/kg BW), and Pd12 (0.10, 0.20, 0.30, 0.40, and 0.50 mg/kg BW) in a pilot study to identify the optimum mifepristone dosage for these time points. The results indicated that mifepristone treatment at doses of 1.20, 0.40, and 0.20 mg/kg BW, respectively, on Pd4, Pd8, and Pd12 were most suitable. As the ducts and alveoli were growing on Pd4 and Pd8, while almost completely developed on Pd12, we designed four strategies to evaluate the effect of mifepristone on mammary gland development. These included Pd4, Pd4 + Pd8, Pd8 and Pd8 + Pd12.

**FIGURE 1:**
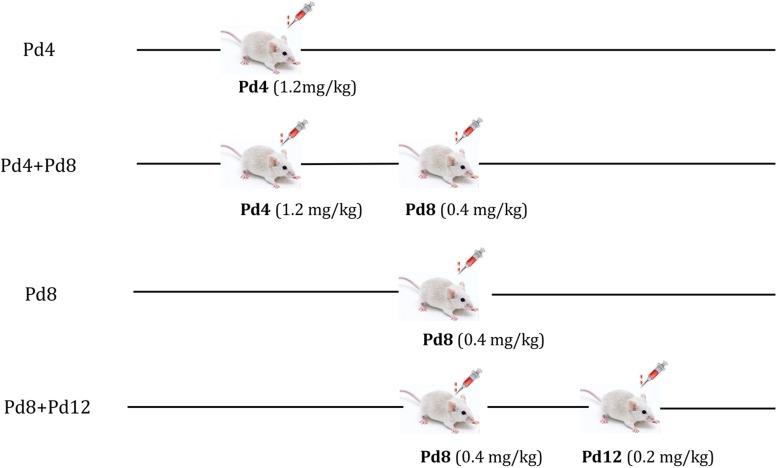
The strategy of mifepristone administration to the pregnant mice on pregnancy day 4 (Pd4), Pd8, or Pd12. Pd4: mice were treated with 1, 3-propanediol or mifepristone on Pd4; Pd4 + Pd8: mice were treated with 1, 3-propanediol or mifepristone on Pd4 and Pd8, respectively; Pd8: mice were treated with 1, 3-propanediol or mifepristone on Pd8; Pd8 + Pd12: mice were treated with 1, 3-propanediol or mifepristone on Pd8 and Pd12, respectively.

Using these strategies, pregnant mice were subjected to mifepristone treatments (Figure 1). Each strategy was an independent experiment. During each strategy, mated female mice with the same pregnancy day were caged together and were randomly divided into a control group and a mifepristone group on the day first being treated with 1, 3-propanediol or mifepristone, respectively. Control mice were treated on the same day with mifepristone mice during each strategy. All treatments were performed at a fixed time of a day (9:00 am–10:00 am). On the day of the experiment, control group were injected subcutaneously with 0.10 mL of only solvent (1, 3-propanediol), while mifepristone group received specific dosage of mifepristone in the same volume of the solvent. Briefly, for Pd4 treatment, control group and mifepristone group were subcutaneously injected with 0.10 mL of 1, 3-propanediol and 1.20 mg mifepristone/kg BW, respectively, on Pd4. For Pd4 + Pd8 treatment, control mice were subcutaneously injected with 0.10 mL of 1, 3-propanediol on Pd4 and on Pd8, respectively, while mifepristone-treated mice were firstly subcutaneously injected with 1.20 mg mifepristone/kg BW on Pd4, followed with a second subcutaneous injection of 0.40 mg mifepristone/kg BW on Pd8. For Pd8 treatment, control group and mifepristone group were subcutaneously injected with 0.10 mL of 1, 3-propanediol, and 0.40 mg mifepristone/kg BW, respectively, on Pd8. For Pd8 + Pd12 treatment, control group were subcutaneously injected with 0.10 mL of 1, 3-propanediol on Pd8 and on Pd12, respectively, and mifepristone-treated mice were subcutaneously injected with 0.40 mg mifepristone/kg BW on Pd8, followed with a second subcutaneous injection of 0.20 mg mifepristone/kg BW on Pd12. Each group had seven experimental replicates during each strategy. On parturition, the litter sizes for dams from control and mifepristone groups were recorded and were compared as in Table 1.

**TABLE 1:** The effect of mifepristone on the litter size. Pd4, mice were treated with 1, 3-propanediol or mifepristone on Pd4; Pd4 + Pd8, mice were treated with 1, 3-propanediol or mifepristone on Pd4 and Pd8, respectively; Pd8, mice were treated with 1, 3-propanediol or mifepristone on Pd8; Pd8 + Pd12, mice were treated with 1, 3-propanediol or mifepristone on Pd8 and Pd12, respectively.

Groups	*n*	Pd4	Pd4 + Pd8	Pd8	Pd8 + Pd12
Control	7	14 ± 2.16	13 ± 1.67	13.67 ± 3.21	13.8 ± 2.95
Mifepristone	5	14 ± 2.44	14.2 ± 2.86	15.8 ± 1.83	11.0 ± 1.58

### Milk Yield Estimation

After parturition, body weights of dams were recorded on Ld1 (control, 44.84 g ± 1.21; mifepristone, 44.63 g ± 1.08) and the dams received food pellets and water *ad libitum*. Each mother was adjusted with 10 pups on Ld1. To preclude the factors, such as heredity and body weight of the litters, which may affect the determination of the milk yields or growth rate of pups, two dams (one from control group, another from mifepristone group) that littered within a 6-h interval were assigned to cross-foster their pups. Briefly, each dam in the control or mifepristone group nursed five of her own young and five from another dam in mifepristone or control group. In addition, comparable mean litter weights (control, 18.24 ± 1.04 g; mifepristone, 18.15 ± 0.61 g) between the control group and mifepristone group, were maintained when we assigned the 10 pups at birth. Milk yields during early lactation (Ld1 to Ld18) were measured using a weigh-suckle-weigh method, slightly modified from previous reports (Simons et al., 1987; Travers et al., 1996). Briefly, the mother was separated from her pups in a separate clean box for 3 h. Then the pups were weighed, allowed to suckle for 1 h, and were weighed again. This procedure was repeated three times each day over a 12 h period (8:00 am–8:00 pm).

The growth rate of the litter from Ld1 to Ld18 was assessed to evaluate the effect of milk yields on pup growth. For this reason, 10 pups were assigned to each mother on Ld1, as described in milk yields determination above. Body weights of pups were measured and recorded daily between 9:00 and 10:00 a.m. and for a total of 18 days, according to previous methods (Knight et al., 1986; Wilde et al., 1992).

### qRT-PCR

The total RNA from mammary gland was prepared using TRIzol reagent and was reverse transcribed into the first strand cDNA using the TransScript First-Strand cDNA Synthesis SuperMix kit (Transgen Biotech, Wuhan, China). Quantitative real-time PCR was performed using a LightCycler 96 Real-Time PCR system (Roche). SYBR Premix Ex TaqTM II was used to detect gene expression of β-casein, E2 receptor α (ERα), P_4_ receptor (PR), PRL receptor (PRLR), GH receptor (GHR), CORT receptor (CORTR), and OT receptor (OTR). Glyceraldehyde-3-phosphate dehydrogenase (GAPDH) served as an internal control (Kobayashi et al., 2017). The primer sequences for each gene are listed in Table 2. To confirm that the primers are amplifying the correct genes, all PCR products were cloned to the PMD18-T vector (TaKaRa, Dalian, China) for sequencing (Tsingke Biological Technology Co., Ltd., Wuhan, China). Transcript levels of these genes in the mifepristone group relative to the control group was quantified with the 2^–Δ^ ^Δ^ ^Ct^ method, where ΔΔCt = ΔCt (mifepristone group) – ΔCt (control group); ΔCt = Ct_targetgene_ – Ct_GAPDH_.

**TABLE 2:** Primer sequences for real-time PCR in mouse mammary glands.

Gene	Accession number	Primers		Product size (bp)
		Forward	Reverse	
β-casein	NM_009972.2	tccagcctattgctcaaccc	aggaggggcatctgtttgtg	121
ERa	NM_007956.5	tgtgccgtgtgcaatgacta	gcaactcttcctccggttct	159
PR	NM_008829.2	ctggctgtcactatggcgtg	cttacgacctccaaggaccat	191
PRLR	NM_011169.5	tgcacttgcttacatgctgc	tggggccactggttttgtag	227
GHR	NM_010284.3	gtttgaccgggattcgtgga	cgttgtctggatctcacccg	217
CORTR	DQ504162.1	tcaaggtttctgcgtcttcaccct	ttccccatcacttttgtttcggtc	232
OTR	NM_001081147.2	tgtgctgcacgcctttcttc	ggcccgtgaagagcatgtag	147
GAPDH	NM_008084	gagcgaccccactaacatc	gcggagatgatgaccctttt	144

### Western Blot Analysis

Mammary tissue was lysed in RIPA lysis buffer (1g:9 mL, Beyotime, Beijing, China) containing 1% phenylmethylsulfonyl fluoride (PMSF) protease inhibitor. Proteins were separated using 12% SDS-PAGE gel and were transferred to 0.45 μm polyvinylidene difluoride (PVDF) membrane (Millipore, Billerica, MA, United States). Membranes were blocked in 5% BSA for 2 h, incubated with primary antibody at 4°C overnight, washed in TBST, incubated with HRP-conjugated secondary antibody at room temperature for 2 h, and were finally visualized through ECL (Beyotime, Beijing, china). Mouse primary anti-β-casein antibody (1:700, Santa Cruz Biotechnology, United States), rabbit polyclonal primary anti-β-actin antibody (1:200, Servicebio, Wuhan, China) and HRP-conjugated goat anti-mouse or -rabbit secondary antibody (1:2000, Servicebio, Wuhan, China) were used. Considering that the use of primary mouse β-casein antibody detected with an anti-mouse secondary antibody in mouse tissue may produce the non-specific background of IgG (25 kD), mammary gland from 8-week old female virgin mice was used as a negative control (Youth) for β-casein expression. There were seven replicates for each group in the western blot assay. Quantification of the integrated optical identity (IOD) was performed by Image-Pro plus 6.0 software (Media Cybernetics, Inc., Silver Spring, MD, United States). The absolute IOD of β-casein was obtained by subtracting IOD of non-specific IgG, from the total IOD of β-casein, and the relative scales were calculated on the basis of β-actin expression.

### Whole-Mounting and Histological Analysis

For whole mount analysis, the fourth mammary gland was excised and spread on glass slides and fixed in Carnoy’s fixative (100% ethanol:chloroform:glacial acetic acid, 6:3:1) overnight at room temperature. They were then washed in 70%, 35% and 15% ethanol for 15 min each, rinsed through a graded series of alcohol followed by a wash in distilled water for 5 min, stained in carmine alum for 6 h, washed in 70, 95, and 100% ethanol for 15 min each, cleared in xylene, and mounted. Branches and alveolar buds were observed under an Olympus SZX 16 stereoscopic microscope (Olympus, Tokyo, Japan). As it was difficult to count the number of ductal branches on Pd7, Pd10, Pd16, and Ld3, and mammary alveoli on Ld3 clearly, we did not analyze them on these days.

For hematoxylin and eosin (H&E) staining, the fourth mammary glands were fixed in 4% neutral polyformaldehyde, washed, dehydrated, cleared and embedded in paraffin. They were cut into 5-μm sections, dewaxed in xylene and rehydrated through a graded series of alcohol, followed by a wash in distilled water for 5 min. The sections were finally stained with H&E (Servicebio, Wuhan, China). The diameters of the alveolar on Ld3 were analyzed with the Image-Pro plus 6.0 software (Media Cybernetics).

### Hormone Analysis

Blood was collected from dams on Pd7, Pd10, and Pd16 at 10:00 am, while on Ld3, blood was collected at 9:00 pm as dams were used for determining milk yields from 8:00 am–8:00 pm. Blood was collected from the retro-orbital sinus of with a pyrogenic- and endotoxin-free tube. Serum was collected through centrifugation and stored at −80°C. Serum E_2_ and P_4_ were assayed with commercially ELISA kits for detecting Human E_2_, P_4_ (Beijing North Institute of Biotechnology Co., Ltd., Beijing, China). Serum PRL, GH, corticosterone (CORT) and OT were measured with Mouse PRL, GH, CORT and OT ELISA kits (Nanjing Jiancheng Bioengineering Institute, Nanjing, China), respectively. For E_2_ and P_4_ detection, intra- and inter-assay coefficients of variation (CV) were <15%. For PRL, GH, CORT and the OT immunoassay, the intra- and inter-assay CVs were <10 and <12%, respectively. Detection limits for E_2_ and P_4_ were ≤40 pg/mL and 0.2 ng/mL, respectively. The detection limits for PRL, GH, CORT, and OT were 2–600 ng/mL, 0.10–30 ng/mL, 0.51–50 ng/mL, and 51–500 pg/mL, respectively.

### Statistics

Data were analyzed using the SPSS 17.0. Differences in milk yields, mammary gland weight, β-casein expression, alveolar size, body weight of pups, hormone concentrations, and hormone receptors mRNA expression between control and mifepristone-treated groups were determined with Student’s *t*-tests. All data were represented as means ± SD. A value of *P* < 0.05 was considered statistically significant.

## Results

### Mifepristone Treatment During Pregnancy Induced Milk Yield Reduction on Early Lactation Days

**FIGURE 2:**
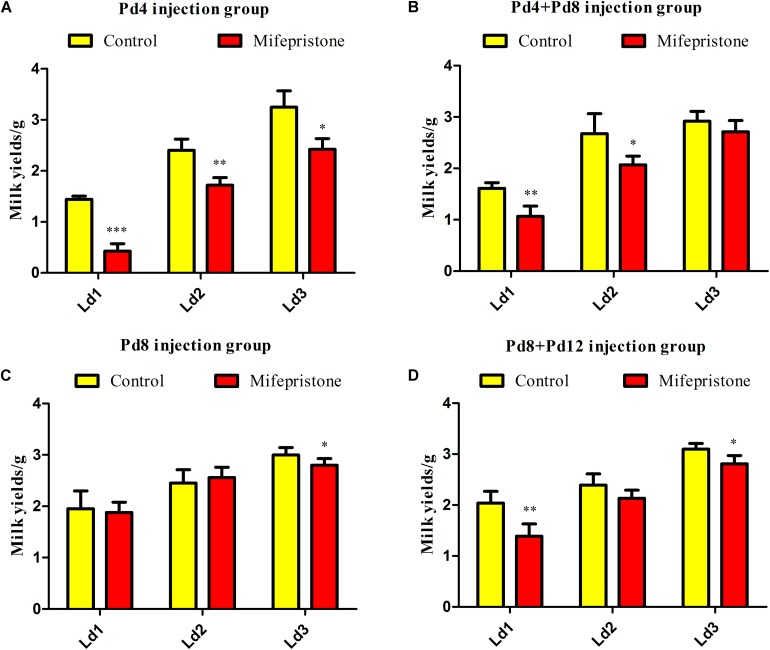
The effect of four different treatments with mifepristone during pregnancy on milk yields. **(A–D)** illustrated the milk yields from lactation day 1 (Ld1) to Ld3 after performing pregnancy day 4 (Pd4), Pd4 + Pd8, Pd8, and Pd8 + Pd12 strategy, respectively. Data are represented as means ± SD, ^∗^indicates the milk yields in mifepristone group are different from that of control group, ^∗^*P* < 0.05, ^∗∗^*P* < 0.01, ^∗∗∗^*P* < 0.001.

Four different strategies were adopted for treating the pregnant mice with mifepristone. There were no differences in the litter sizes of dams between control group and mifepristone group in each strategy (Table 1). Milk yields were calculated on Ld1, Ld2, and Ld3 after parturition. As shown in Figure 2A, Pd4 treatment caused a decrease in milk yields of the mifepristone group on Ld1, Ld2 and Ld3 (*P* < 0.0001, *P* = 0.003, and *P* = 0.015, respectively), compared with those from control group. In the Pd4 + Pd8 strategy, milk yields from the mifepristone group were less than those from the control group on Ld1 and Ld2 (*P* = 0.001 and *P* = 0.013, respectively), but no different in milk yield was found between two groups on Ld3 (Figure 2B). In the Pd8 strategy, mifepristone-treated mice showed no change in milk yields on Ld1 and Ld2, but exhibited a significant reduction on Ld3 (*P* = 0.04) compared to those from the control group (Figure 2C). In the Pd8 + Pd12 strategy, milk yields of the mifepristone group were less on Ld1 and Ld3 than (*P* = 0.002 and *P* = 0.01, respectively), but on Ld2, the milk yields were comparable with those in the control group (Figure 2D).

In these four strategies, milk production reduced on Ld1, Ld2, and Ld3 in the mifepristone-treated group from the Pd4 strategy, whereas in the other three strategies, this occurred only on one or two of these days after mifepristone treatment. Owing to its better efficacy in decreasing milk production for three consecutive days after parturition, we selected the Pd4 strategy for subsequent experiments.

### Mifepristone Administration on Pd4 Decreased Mammary Gland Weight, Milk Yields and Pup Growth During Pregnancy or Early Lactation

**FIGURE 3:**
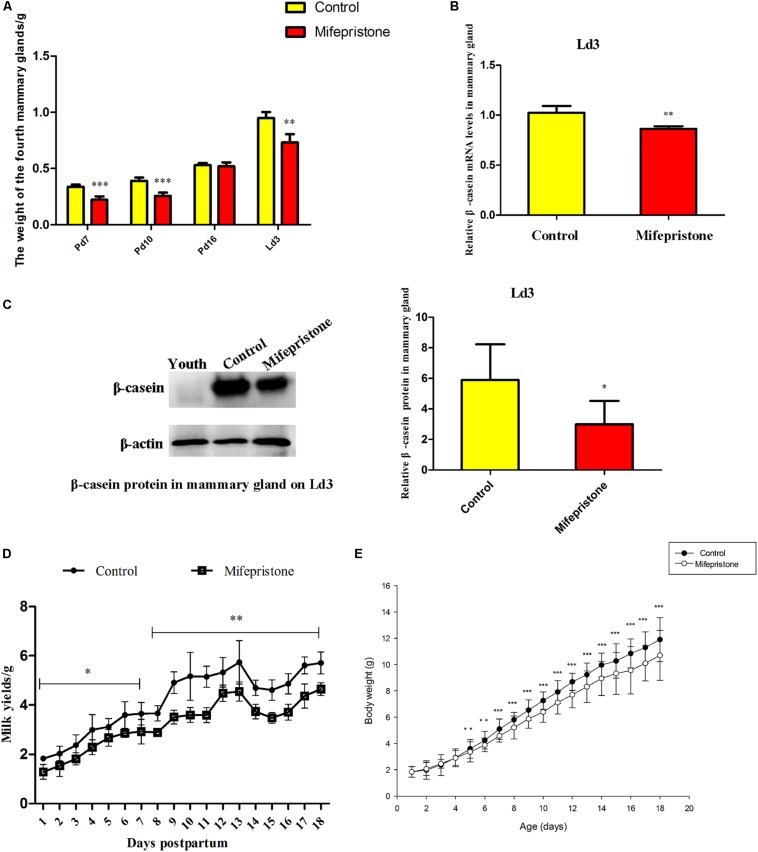
Effects of mifepristone administration on pregnancy day 4 (Pd4) on mammary glands weight, β-casein expression, milk yields and litter growth rates during pregnancy and early lactation. **(A)** The weight of the fourth mammary glands on Pd7, Pd10, Pd16, and lactation day 3 (Ld3) after mifepristone administration. **(B)** qRT-PCR analysis of β-casein mRNA on Ld3. This experiment was repeated at least three times. **(C)** Western blot analysis for β-casein expression on Ld3. The evaluation of β-casein relative to β-actin in two groups were calculated with integrated optical identity (IOD). **(D)** Milk yields of dams from Ld1 to Ld18 after parturition, determined via weigh-suckle-weigh method. **(E)** Body weight gain in pups from Ld1 to Ld18. Data are represented as means ± SD, ^∗^indicates the difference between mifepristone group and control group, ^∗∗^*P* < 0.01, ^∗∗∗^*P* < 0.001.

The weight of mammary glands in mice from the mifepristone group decreased on Pd7, Pd10, and Ld3 (*P* < 0.0001, *P* < 0.0001, and *P* = 0.009, respectively), compared to that of controls (Figure 3A). However, on Pd16, this difference was not seen. Mifepristone administration caused a significant decrease in both mRNA and protein levels of β-casein (*P* = 0.003 and *P* = 0.018, respectively), in mice mammary glands on Ld3, compared to those in solvent controls (Figures 3B,C). From Ld1 to Ld18, mice in the mifepristone group produced less milk than those in the control group (*P* < 0.05) (Figure 3D). Further, from Ld5 to Ld18, pups weight gain in the mifepristone group was lower than that of the control (*P* < 0.01); However, this difference in both groups was not seen from Ld1 to Ld4 (Figure 3E).

### Mifepristone Administration on Pd4 Affected Mammary Alveoli Formation During Pregnancy and Early Lactation

**FIGURE 4:**
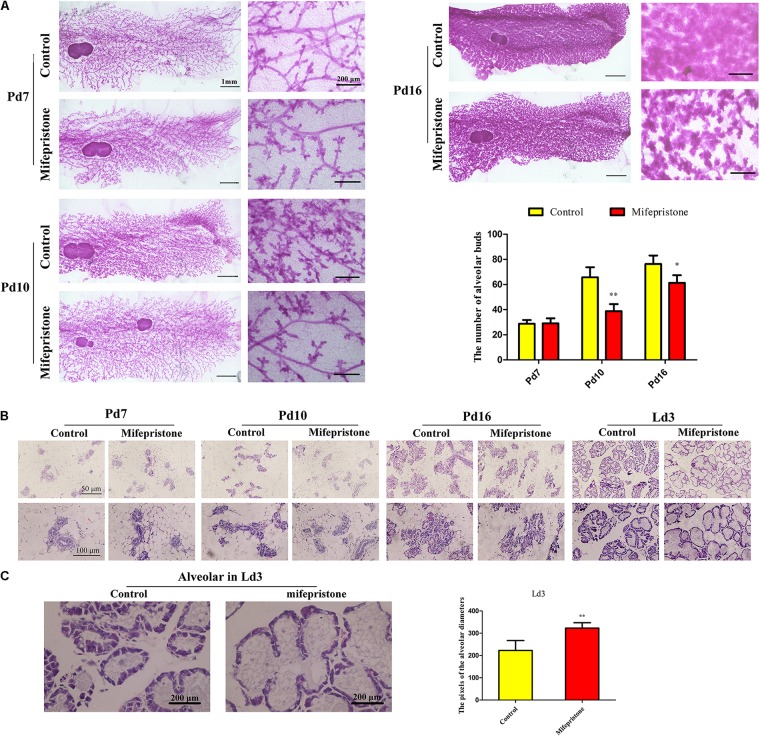
The effect of mifepristone administration on pregnancy day 4 (Pd4) on mammary branching and alveoli formation. **(A)** Whole-mount staining of mammary glands of mice on Pd7, Pd10, and Pd16. Quantification of the number alveolar buds in mammary glands of controls and mifepristone-treated mice. **(B)** Hematoxylin and eosin (H&E) staining of mice mammary glands on Pd7, Pd10, Pd16, and lactation day 3 (Ld3). **(C)** The quantification of alveolar diameters on Ld3. Data are represented as means ± SD, ^∗^indicates the difference between mifepristone group and control group,^∗^*P* < 0.05, ^∗∗^*P* < 0.01.

Whole-mount analysis showed that there was no difference in the number of alveolar buds between the control and mifepristone groups on Pd7 (Figure 4A), whereas on Pd10 and Pd16, the number of alveolar buds reduced in mifepristone-treated mice compared to controls (*P* = 0.009 and *P* = 0.044, respectively) (Figure 4A).

No difference was found in the morphology of alveoli between two groups on Pd7 and Pd10 as seen in H&E staining (Figure 4B). However, on Pd16, the alveoli in the mifepristone-treated mice were disorganized compared with those from controls (Figure 4B). On Ld3, histology of the mammary glands showed an enlarged alveolar lumen within which a few cells were disseminated in mifepristone-treated mice as compared to the control group (*P* < 0.05) (Figures 4B,C).

### Mifepristone Administration on Pd4 Altered Hormone Levels During Pregnancy and Early Lactation

**FIGURE 5:**
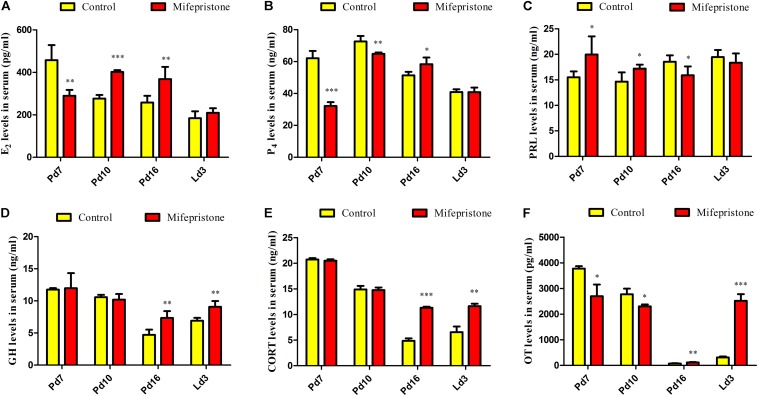
Effects of mifepristone treatment on pregnancy day 4 (Pd4) on serum hormones levels of mice on Pd7, Pd10, Pd16, and lactation day 3 (Ld3). **(A)** Serum E_2_ levels. **(B)** Serum P_4_ levels. **(C)** Serum PRL levels. **(D)** Serum GH levels. **(E)** Serum CORT levels. **(F)** Serum OT levels. Data are represented as means ± SD, ^∗^indicates the difference between mifepristone group and control group, ^∗^*P* < 0.05, ^∗∗^*P* < 0.01, ^∗∗∗^*P* < 0.001.

As shown in Figure 5A, on Pd7, concentrations of E_2_ in the mifepristone group decreased significantly (*P* = 0.001), and then showed an increase on Pd10 and Pd16 (*P* < 0.0001 and *P* = 0.005, respectively), but did not change on Ld3, compared to controls (Figure 5A). Compared to controls, P_4_ levels in mifepristone group showed a reduction on Pd7 and Pd10 (*P* < 0.0001 and *P* = 0.001, respectively), but rose on Pd16 (*P* = 0.011). However, PRL concentrations in the mifepristone group increased on Pd7 and Pd10 (*P* = 0.047 and *P* = 0.02, respectively), but decreased on Pd16 (*P* = 0.048) compared to those in the control group. On Ld3, there was no difference in P_4_ or PRL concentrations between two groups (Figures 5B,C). Both GH and CORT levels in the mifepristone group remained steady on Pd7 and Pd10, but increased on Pd16 and Ld3 compared to those in the control group (*P* < 0.01) (Figures 5D,E). Levels of OT in the mifepristone-treated mice were lower on Pd7 and Pd10 (*P* = 0.016 and *P* = 0.025, respectively), but were higher on Pd16 and Ld3 (*P* = 0.005 and *P* < 0.0001, respectively), compared to those in controls (Figure 5F).

### Mifepristone Administration on Pd4 Altered Mammary mRNA Expression of Hormonal Receptors During Pregnancy or Early Lactation

**FIGURE 6:**
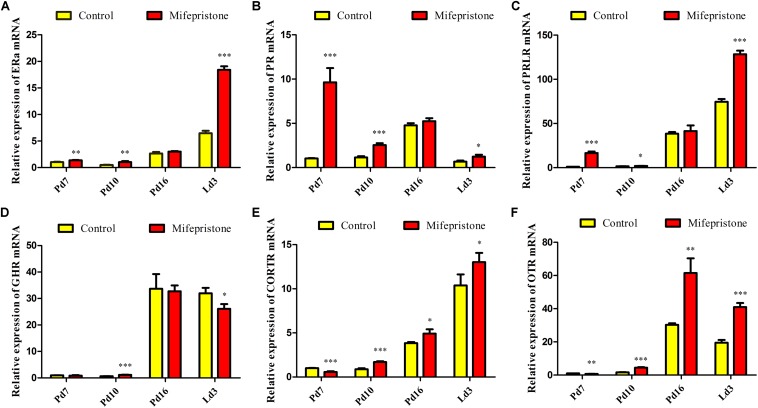
Effect of mifepristone treatment on pregnancy day 4 (Pd4) on mammary mRNA expression of hormone receptors on Pd7, Pd10, Pd16, and lactation day 3 (Ld3). **(A)** ERα mRNA expression. **(B)** PR mRNA expression. **(C)** PRLR mRNA expression. **(D)** GHR mRNA expression. **(E)** CORTR mRNA expression. **(F)** OTR mRNA expression. These experiments were repeated at least three times. Data are represented as means ± SD, ^∗^indicates the difference between mifepristone group and control group, ^∗^*P* < 0.05, ^∗∗^*P* < 0.01, ^∗∗∗^*P* < 0.001.

The ERa mRNA expression in mifepristone group increased on Pd7, Pd10, and Ld3 (*P* = 0.005, *P* = 0.006, and *P* < 0.0001, respectively), when compared to that in the control group; However, on Pd16, this difference disappeared between two groups (Figure 6A). Similarly, on Pd7, Pd10, and Ld3, the PR and PRLR mRNA levels increased in the mifepristone group (*P* < 0.05) compared to those in controls, but again on Pd16, this difference vanished (Figures 6B,C). The GHR mRNA levels in the mifepristone group increased on Pd10 (*P* = 0.001) and decreased on Ld3 (*P* = 0.021), but remained unchanged on Pd7 and Pd16 compared to that in controls (Figure 6D). However, the CORTR mRNA expression in the mifepristone group decreased on Pd7 (*P* = 0.001), but increased on Pd10, Pd16, and Ld3 (*P* = 0.001, *P* = 0.022, and *P* = 0.049, respectively), as compared to that in controls (Figure 6E). Similarly, mifepristone treatment caused a reduction in the OTR mRNA expression on Pd7 (*P* = 0.005), but an increase on Pd10, Pd16, and Ld3 (*P* < 0.0001, *P* = 0.004, and *P* < 0.0001, respectively), compared to that in controls (Figure 6F).

## Discussion

The current study demonstrated that mifepristone treatment on mice with early pregnancy decreased milk yields, reduced β-casein expression, curtailed the growth rate of litters, enlarged the alveolar lumen, and altered the serum levels of E_2_, P_4_, PRL, GH, CORT, OT as well as their receptors during pregnancy and lactation, indicating the mammary gland dysplasia and postpartum hypogalactia in the murine model. Previous studies demonstrated the effect of mifepristone as an anti-progestins on PRL release and on specific gene expression during mammary gland development and lactation in mice or rats *in vitro* (Shiqing et al., 1990; Li et al., 1995), or *in vivo* (Deis et al., 1989; Jahn et al., 1991; Michna et al., 1991; López-Fontana et al., 2012). However, the current study showed differences in two main points. Firstly, though postpartum hypogalactia is still considered a major problem in human health and in the livestock breeding industry, the researches regarding its mechanisms are rarely reported. Here, we aim to explore the mechanisms of postpartum hypogalactia by mimicking mammary gland development and secretion during pregnancy or lactation after several hormones disturbed by mifepristone. Secondly, by carrying out the study *in vivo*, we have tracked milk yields, weight of the mammary glands, ducts, alveolar, and growth rate of pups as well as the changes in the six hormones (E_2_, P_4_, PRL, GH, CORT, and OT) and their receptors, which are closely related with mammary gland growth and lactation from pregnancy to lactation.

Mifepristone is an effective drug for termination of early pregnancy. Previous reports suggested that mifepristone administration at doses of 1.25–2.50 mg/kg BW on Pd4 had no impact on the reproduction of mice (Huang et al., 2005; Lv et al., 2012; Chen et al., 2015; Yang et al., 2016). However, mifepristone administration at doses of 0.30–2.00 mg/kg BW at approximately Pd8.5 caused 60–100% of abortions in mice (Szekeres-Bartho et al., 1997; Lv et al., 2012). Mifepristone treatment at doses of 0.40–12.50 mg/kg BW during late pregnancy (Pd14–Pd19) induced preterm labor in mice, with abortion rates of 66–100%. These results demonstrate that the dosage and the application time of mifepristone during pregnancy are closely associated with abortion rates. Here, we applied 1.20, 0.40, and 0.20 mg mifepristone/kg BW to mice on Pd4, Pd8, and Pd12, respectively, and we found that these strategies did not affect pregnancy rates and litter sizes of mice. However, the efficacy of these four strategies were different to some extents in reducing milk yields; mifepristone administration on early pregnancy (Pd4) rather than that on middle or late pregnancy (Pd8 or Pd12) was more efficient in decreasing the milk yields from Ld1 to Ld3.

There are four main techniques for measuring milk yields in mice. These include direct milking, the weigh-suckle-weigh method, isotope transfer from mother to the young via milk, and isotope dilution (Knight et al., 1986). The disadvantages of the direct milking method are that it relies on the ability of milkers to empty the glands, and that exogenous OT injection and milking operation may easily induce stress, which inhibited the milk ejection reflex in mice (DePeters and Hovey, 2009). While the isotope transfer has its difficulty in controlling the appropriate activity of isotope when measured. The isotope dilution technique is also complicated since it contains the recycling of the isotope through the mother drinking the pup’s urine (Knight et al., 1986). For decades, the weigh-suckle-weigh method has been more acceptable for its simple and effective evaluation of milk volume in mice (Boston et al., 2001; Liu et al., 2017). This method is also widely used in ruminants such as in goats or cattle (Mummed, 2012; Hogberg et al., 2016). However, the disadvantage of this weigh-suckle-weigh method is that milk secretion may be reduced after a separation period over 4 h (Hanwell and Linzell, 1972). To avoid this problem, the separation and the suckling periods were set as 3 and 1 h, respectively, in the present study, and for a total of 12 h during a day. Moreover, the reduction in β-casein expression and pup weight gain during early lactation in mifepristone group mice further confirmed the milk insufficiency verified by weigh-suck-weigh method. Nevertheless, the amount of milk produced by each mouse was previously reported to be proportional to lactation numbers, litter size, and maternal body weight (Knight et al., 1986). To avoid these factors, all the mice used for this study were 8 weeks old and with similar body weight (31 ± 1 g) on Pd1.

Mammary gland development and lactation are tightly regulated by hormones. Upon pregnancy, P_4_ and PRL initiate a proliferation drive for secondary and tertiary ductal branching and alveolar morphogenesis. In the latter stages of pregnancy, approximately Pd18 in mice, CORT and OT are required for individual alveoli differentiation from alveolar buds into the secretory activation phase, in preparation for postpartum secretion (Traurig, 1967; Brisken et al., 1998; Nguyen et al., 2001). Thus, the decrease of the weight of the mammary gland in mifepristone group on Pd7, Pd10, and Ld3 may be a comprehensive effect of alterations in P_4_, PRL, CORT, and OT profiles, whereas the absence of difference in mammary gland weight on Pd16 between two groups may be caused by preparation for parturition, long-term effect of mifepristone on mammary growth and milk components in late pregnancy in mice or rats (Cole, 1933; Deis et al., 1989; López-Fontana et al., 2012). In addition, in the current study, though it was difficult to count the secondary and tertiary ductal branches of mammary glands during late pregnancy and lactation, we observed an obvious decrease in alveolar buds and enlarged alveolar lumina after mifepristone treatment. Furthermore, an enlarged alveolar lumen after mifepristone administration is in accordance with a previous report, showing that that loss of mammary gland development associated gene *Numb or Numbl/l*, leading to an enlarged lumen of alveoli and failure in lactation during pregnancy in mice (Zhang et al., 2016).

Mifepristone decreased P_4_ or E_2_ levels on Pd7, these results were in agreement with previous reports, in which P_4_ or E_2_ levels declined two days after mifepristone administration in humans or mice (Somell et al., 1990; Chen et al., 2015). However, subsequent increase of E_2_ concentrations on Pd10 and Pd16, and of P_4_ concentrations on Pd16 after mifepristone administration may be attributed to ovarian response as reported in humans (Somell et al., 1990). Studies have reported that E_2_, P_4_ or CORT affected PRL levels in rats (Meites and Nicoll, 1966; Caligaris et al., 1974), and this may explain the reason why PRL increased on Pd7 and Pd10, but decreased on Pd16. Increased P_4_ levels and the decreased PRL levels on Pd16 in mice with reduced milk yields were consistent with reports in low-colostrum-producing sows (Foisnet et al., 2010). In our study, CORT and GH levels did not change during early pregnancy, but rose on Pd16 and Ld3 after mifepristone treatment; effects of these enhanced concentrations of GH and CORT on late pregnancy or lactation may be due to self-regulation in mice, or caused by the suckling stimulation by pups in mifepristone-treated mice. These results were also consistent with clinical reports in which the concentrations of cortisol increased in sows with PDS, compared to those in PDS negative sows (Kaiser et al., 2018), and that increase in GH and CORT levels increased the milk yields of cows or rats after prolonged lactation, and suckling was reported to increase plasma GH levels in rats (Machlin, 1973; Miki et al., 1981; Flint et al., 1984). Synthetic glucocorticoids (prednisolone or dexamethasone) treatment raised plasma glucose and caused a decrease in milk production in lactating cows (Maplesden et al., 1960; Hartmann and Kronfeld, 1973; Kronfeld and Hartmann, 1973). However, the glucose uptake and utilization of the mammary gland were diminished after glucocorticoids administration, which directly resulted in the reduction in milk yields (Hartmann and Kronfeld, 1973). α-Lactalbumin is a milk whey protein that can modify the substrate specificity of galactosyltransferase to include glucose, thus enhancing the synthesis of lactose and the milk production (Brodbeck et al., 1967; Brew et al., 1968; Stacey et al., 1995). However, high dose administration of cortisol in rats and mouse mammary gland explants caused a lower lactose content or inhibition of α-lactalbumin accumulation (Ono and Oka, 1980; Llopis et al., 1990). Therefore, in the current study, the increases in CORT and CORTR mRNA levels on Pd16 and on Ld3 may lead to inhibition of α-lactalbumin accumulation, glucose uptake, and lactose synthesis in mammary gland of mifepristone-treated mice, which finally caused a decrease in milk production. In addition, although decreased OT levels on Pd7 and Pd10 may be directly induced by mifepristone, increased levels of OT on Pd16 and Ld3 may also be caused by the suckling stimulation of litters to induce greater milk ejection from mifepristone-treated mice (Bruckmaier and Blum, 1996).

Nevertheless, basal concentrations of the hormone levels may be different from the previous reports in mice or rats (McCormack and Greenwald, 1974; Parkening et al., 1978; Higuchi et al., 1986), these may have been caused by different detection methods (radioimmunoassay or ELISA), different species or different strains of mice, or different bleeding methods (Sinha et al., 1972). In conclusion, mifepristone treatment during early pregnancy caused alterations in serum levels of E_2_, P_4_, PRL, GH, CORT and OT levels as well as in the mRNA expression profiles of their receptors during pregnancy or lactation. This may lead to mammary gland dysplasia and postpartum hypogalactia in mice. However, hormones exert their function mainly through their receptors. The defects in mammary gland development and hypogalactia after mifepristone treatment may be a comprehensive regulation of E_2_, P_4_, PRL, GH, CORT, OT, hormone receptors, and hormone-related signaling pathways. The current murine model offers a platform for further exploration of the mechanisms underlying milk insufficiency on early lactation days in mammals.

## Data Availability Statement

All datasets generated for this study are included in the article/supplementary material.

## Ethics Statement

The animal study was reviewed and approved by the Institutional Animal Care and Use Committee of the Huazhong Agricultural University.

## Author Contributions

MD, HZ, JC, and LH designed the research. HZ, MR, LY, and XJ performed the experiments. HZ and YD analyzed the data. HZ wrote the manuscript.

## Conflict of Interest

The authors declare that the research was conducted in the absence of any commercial or financial relationships that could be construed as a potential conflict of interest.
